# Systemic Aspergillosis: Radiological Findings in a Case With Diffuse Large B Cell Lymphoma Treated by Ibrutinib

**DOI:** 10.7759/cureus.19911

**Published:** 2021-11-26

**Authors:** Umur Anil Pehlivan, Hasan Bilen Onan, Ertugrul Bayram, Celalet Keser, Semra Paydas

**Affiliations:** 1 Radiology, Van Baskale State Hospital, Van, TUR; 2 Radiology, Çukurova University Faculty of Medicine, Adana, TUR; 3 Medical Oncology, Çukurova University Faculty of Medicine, Adana, TUR; 4 Anesthesiology and Reanimation, Çukurova University Faculty of Medicine, Adana, TUR

**Keywords:** adverse event, chest imaging, neuroimaging, systemic aspergillosis, ibrutinib, diffuse large b-cell lymphoma

## Abstract

Herein, we present a case of systemic aspergillosis with a fatal outcome in a case with diffuse large B cell lymphoma (DLBCL) treated by ibrutinib. Aspergillosis was suspected clinically and proven microbiologically. Radiological findings were compatible with aspergillosis. We aim to review radiological findings in a case with DLBCL treated with ibrutinib, which is an important tyrosine kinase inhibitor used in lymphoid neoplasias.

## Introduction

Ibrutinib is a potent inhibitor of Bruton’s tyrosine kinase (BTK), and therefore, it offers a long-term survival advantage when used for the treatment of B-cell malignancies [1]. However, some case reports have identified side-effects associated with ibrutinib therapy, including systemic fungal infections [2-4]. Aspergillosis is a mycotic infection caused by *Aspergillus fumigatus* that mainly affects the pulmonary system [5]. In immunocompromised patients, *Aspergillus* may cause systemic, invasive, life-threatening complications in the presence of fungemia. Aspergillosis presents with specific radiological findings, but not in all cases [5,6]. Early diagnosis and treatment are crucial for immunocompromised patients because otherwise, the infection rapidly progresses, and irreversible/catastrophic events occur with poor outcomes.

## Case presentation

**Figure 1:**
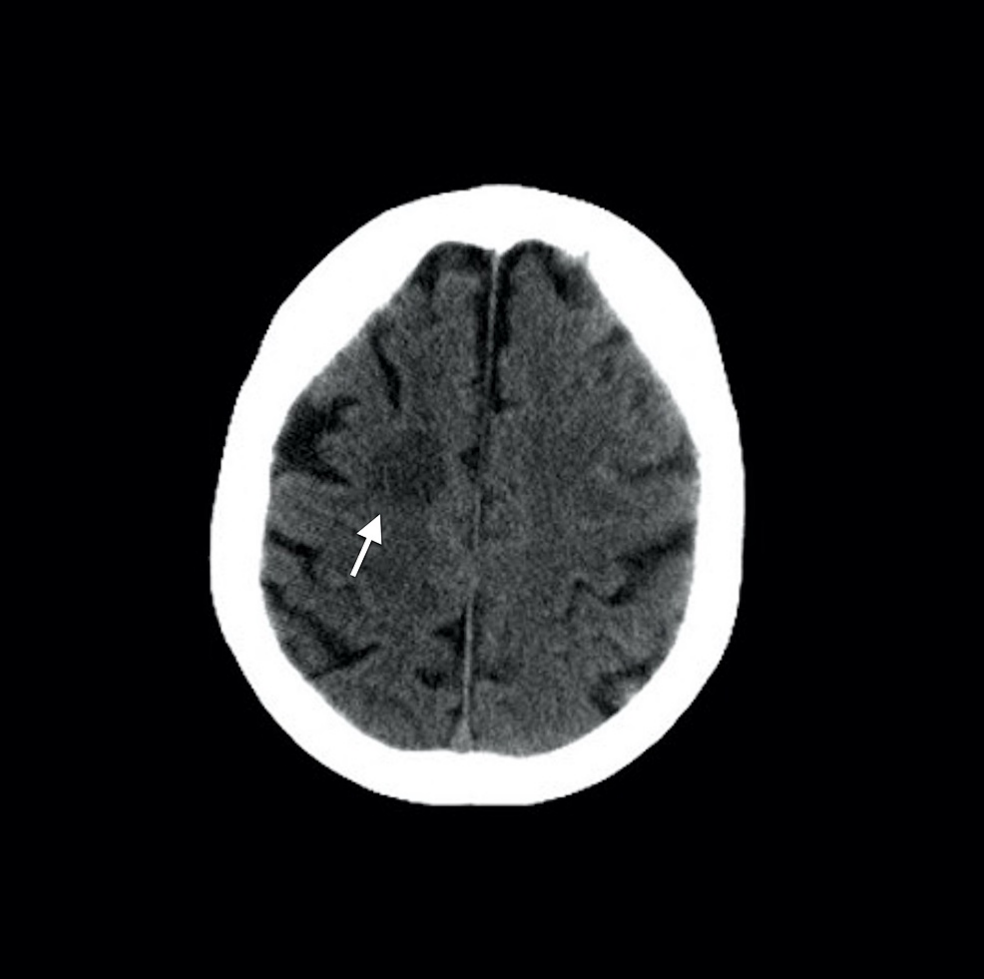
Hypodense nodular lesion with edema in right frontal region is present in non-contrast enhanced computed tomography.

**Figure 2:**
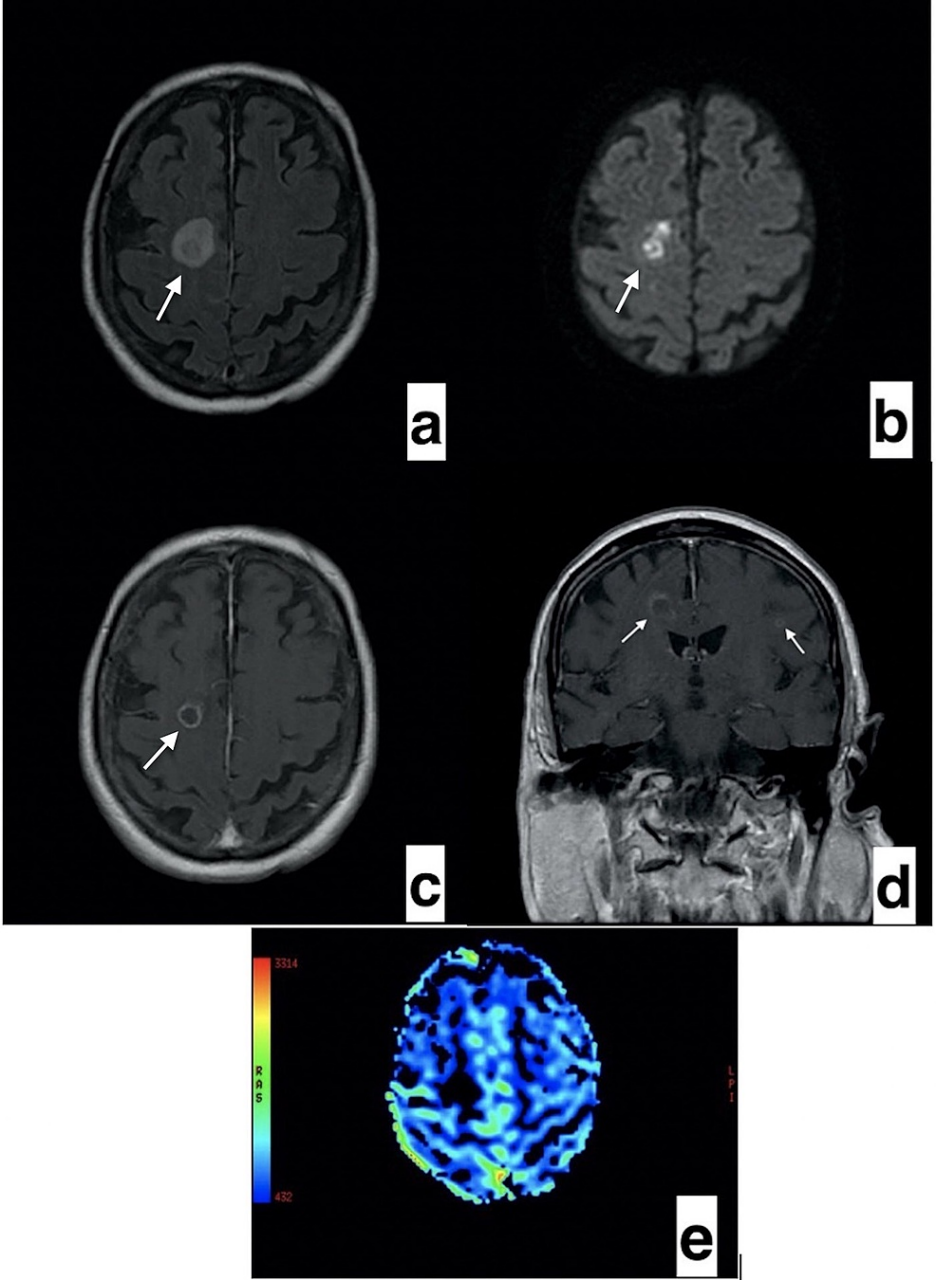
Bilateral nodular lesions with edema around the lesion (a), peripheral restricted diffusion (b), and peripheral enhancement (c, d), were observed. These lesions were hypoperfused in perfusion-weighted images (e).

**Figure 3:**
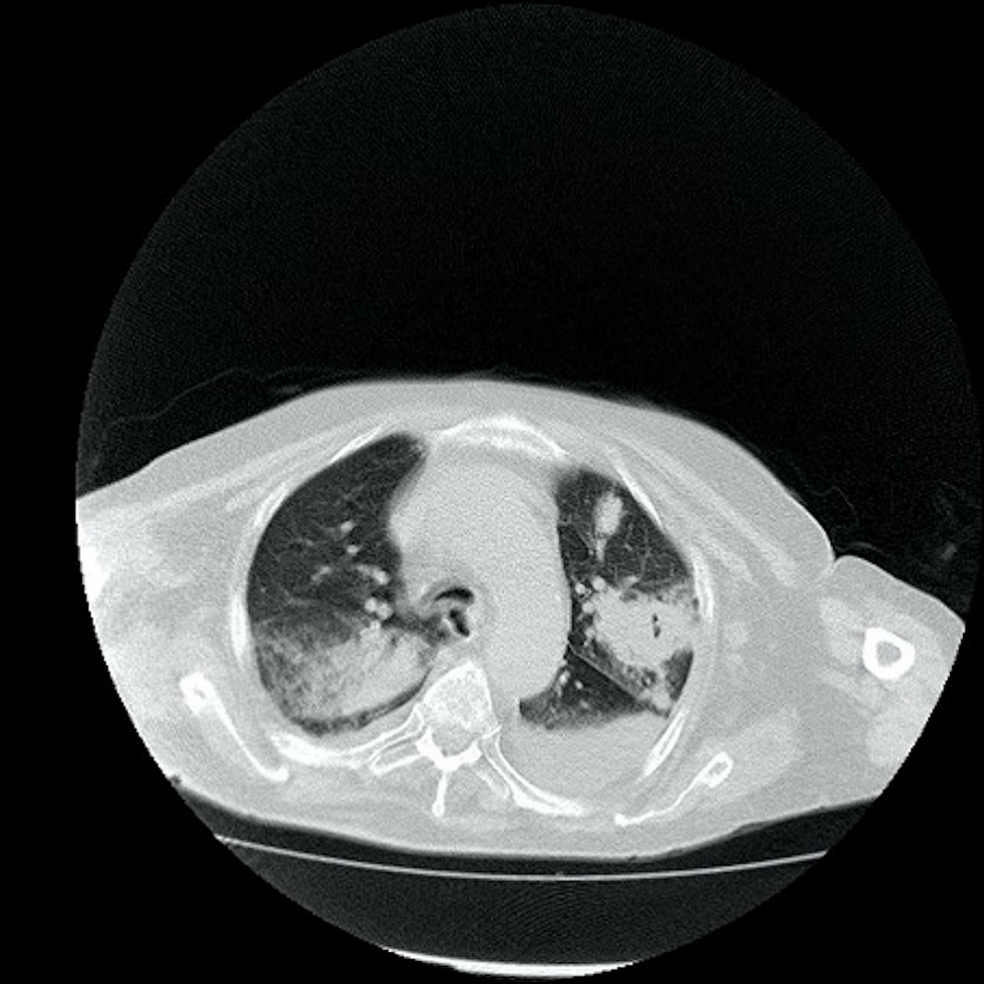
Bilateral patchy ground-glass opacities and consolidation were observed in thorax computed tomography.

In March 2014, an 80-year-old male presented with resistant diffuse large B-cell lymphoma (DLBCL; activated B-cell type). The patient was treated with rituximab-mini CHOP (cyclophosphamide, doxorubicin, vincristine, prednisolone). In May 2019, the patient relapsed and was treated with R-bendamustine and lenalidomide. In March 2020, the patient relapsed again and was treated with ibrutinib. Initially, no significant toxicity developed, although the patient suffered muscle pains. However, after three months of ibrutinib treatment, the disease progressed. After progression, venetoclax was started at 20 mg/day with a ramp-up schedule while ibrutinib was continuing. However, one week later, Babinski positivity, tachycardia, arrhythmia, respiratory distress, and disorientation developed. Non-contrast cerebral computed tomography (CT) showed a hypodense nodular lesion with edema in the right frontal region (Figure 1). First, cerebral lymphoma involvement was suspected; however, magnetic resonance imaging (MRI) with conventional and perfusion imaging showed bilateral nodular lesions with edema around the lesion, peripherally restricted diffusion, and peripheral enhancement. These lesions were hypoperfused in perfusion-weighted images (Figure 2). All these radiologic findings were compatible with opportunistic infections. Bilateral patchy ground-glass opacities, consolidation, and left pleural effusion were observed on thorax CT (Figure 3). These radiological images were compatible with opportunistic lung infection. A serum *Aspergillus* antigen (galactomannan) test was positive. When the diagnosis of aspergillosis was microbiologically proven, neutropenia was not detected. Then, amphotericin B and meropenem were started. However, three days later, multisystem organ failure developed. Hemodiafiltration for acute renal failure and intravenous immunoglobin for hypogammaglobulinemia was administered; despite these interventions, the patient died on the eighth day. 

## Discussion

Ibrutinib is a BTK inhibitor for B-cell malignancies. However, it may cause invasive fungal infections within the first three months of therapy [7]. In the present case, the pathogenesis of the invasive fungal infection was related to the immunodeficiency due to lymphoma, ibrutinib use, and probably lenalidomide use in previous treatment. The pathogenesis of opportunistic infections due to ibrutinib use is associated with macrophage dysfunction and degradation of the BTK/nuclear factor kappa-B pathway, which may be fatal [8]. Invasive fungal infections of the central nervous system (CNS) occur with hematogenous spreading, cerebrospinal fluid seeding, and adjacent invasion [6]. Aspergillosis accounts for most invasive fungal infections, as in our case. Pulmonary aspergillosis includes five subtypes: aspergilloma, bronchopulmonary aspergillosis, semi-invasive aspergillosis, airway invasive aspergillosis, and angio-invasive aspergillosis [5,9]. Pulmonary manifestations of invasive aspergillosis are due to the invasion of vessels and airways by *A. fumigatus* in immunocompromised patients, as in the present case [5,9]. Multisystemic aspergillosis associated with ibrutinib use may cause catastrophic outcomes. Therefore, the early detection of pathogenic agents is important. Laboratory tests including blood, phlegm, and bronchoalveolar lavage are not always diagnostic. Galactomannan is the only polysaccharide antigen characterized in *A. fumigatus*, and it is useful for the diagnosis of aspergillosis in patients with hematological malignancy [9]. In addition, the radiological features of invasive fungal infections are often nonspecific, but some imaging findings may be diagnostic [5,6]. Therefore, thin-section CT and cerebral MRI should be used for early detection of findings compatible with invasive fungal infections in immunocompromised patients [6,10]. Pulmonary manifestations of invasive aspergillosis can be identified by thin-section CT with specific but non-pathognomonic findings [5,6]. Pulmonary manifestations of invasive aspergillosis are identified by thin-section CT as a tree in bud, ground-glass opacity or pleural-based, wedge-shaped areas of consolidation, peribronchial and lobar consolidation, bronchial dilatation, mucoid impaction, or post-obstructive atelectasis [5]. The MRI features of CNS aspergillosis include perilesional edema, heterogeneous or ring-reduced diffusion, and weak-ring enhancement, as in our case [6]. Some cases of angioinvasive aspergillosis present with multifocal hemorrhagic lesions or stroke [6]. It is also important to know the specific radiological findings of other pathogens involved in the differential diagnosis of aspergillosis [6]. For example, cryptococcus is characterized by the gelatinous transformation in the basal ganglia. Mucormycosis shows restricted diffusion in the frontal lobe. Candidiasis is characterized by microabscesses at the corticomedullary junction, basal ganglia, and cerebellum. Blastomycosis and histoplasmosis present with parenchymal abscesses and meningitis [6]. In addition to conventional MRI, perfusion-weighted MRI can be useful for the differential diagnosis of space-occupying lesions.

## Conclusions

In conclusion, clinicians should be careful when dealing with invasive fungal infections in cases treated by ibrutinib, and treatment must be started as soon as possible to prevent fatal outcomes. Radiologic findings are helpful to detect opportunistic infections in cases treated by ibrutinib. This case report has been presented as an E-Poster at the 46^th^ Turkish National Haematology Congress - October 28-31, 2020.
